# Follicle-stimulating hormone inhibits apoptosis in ovarian cancer cells by regulating the OCT4 stem cell signaling pathway

**DOI:** 10.3892/ijo.2013.2054

**Published:** 2013-08-06

**Authors:** ZHENBO ZHANG, YAPING ZHU, YUNLI LAI, XIAOMEI WU, ZHENGZHONG FENG, YINHUA YU, ROBERT C. BAST, XIAOPING WAN, XIAOWEI XI, YOUJI FENG

**Affiliations:** 1 Department of Obstetrics and Gynecology, Shanghai Jiao Tong University Affiliated First People’s Hospital, Shanghai, P.R. China;; 2 Department of Biochemistry and Molecular Biology, Guangxi Medical University, Nanning, P.R. China;; 3 Department of Pathology, Shanghai Jiao Tong University Affiliated First People’s Hospital, Shanghai, P.R. China;; 4 Department of Experimental Therapeutics, The University of Texas, M.D. Anderson Cancer Center, Houston, TX 77030, USA;; 5 Department of Gynecology, Obstetrics and Gynecology Hospital of Fudan University, Shanghai, P.R. China

**Keywords:** apoptosis, follicle stimulating hormone, OCT4, ovarian epithelial tumor

## Abstract

OCT4, a stem cell marker, is overexpressed in several types of human cancer and can induce resistance to chemotherapy and inhibition of apoptosis. We previously demonstrated that human follicle stimulating hormone (FSH) can inhibit ovarian cancer cell apoptosis. However, the role of OCT4 in FSH-induced inhibition of apoptosis has not been reported in detail. Here, we profiled OCT4 protein expression in ovarian epithelial cancer (OEC) with benign cystadenoma, borderline tumor and carcinoma tissues as well as different ovarian cancer cell lines and normal ovarian epithelial cells. Furthermore, the effects of FSH on OCT4 expression and related signaling pathways were evaluated. The overexpression of OCT4 in ovarian carcinoma and OEC cell lines suggest that OCT4 plays a critical role in OEC carcinogenesis. Moreover, FSH-induced apoptosis inhibition was confirmed and FSH stimulation induced the expansion of CD44^+^CD117^+^ cells with a stem cell-like phenotype. Re-expression of OCT4 enhanced the expression of Notch, Sox2 and Nanog molecules that play critical roles in cancer stem cell proliferation and differentiation. FSH upregulated the expression of Notch, Sox2 and Nanog and these effects were abolished by knocking down OCT4, suggesting that several cancer stem cell pathways are involved in FSH regulation. We also examined OCT4 expression in surgical specimens of ovarian cancer. Immunohistostaining revealed that OCT4 expression was increased in ovarian carcinoma compared with benign cystadenomas and borderline tumors, and OCT4 expression was significantly correlated with histological grade. Staining for OCT4 was increased in serous cystadenocarcinoma, when compared with clear cell carcinoma. In summary, the OCT4 cancer stem cell signaling pathway may mediate FSH-induced inhibition of apoptosis and could provide a target for treatment of ovarian cancer.

## Introduction

Ovarian cancer is one of the most lethal malignancies of the female reproductive system with an overall 50% mortality rate. The high mortality rate of ovarian cancer is largely due to occult metastases within the peritoneal cavity and the advanced stage at detection. More than 90% of ovarian cancers are thought to arise from ovarian surface epithelium (OSE). Epidemiologic studies indicate that gonadotrophin is likely to increase the risk of ovarian cancer, especially in women at the post-menopause stage or under certain pathological conditions. Thus, one emerging theory proposes that the development of ovarian epithelium cancer (OEC) involves gonadotropins. Elevated gonadotropins, follicle stimulating hormone (FSH) and luteinizing hormone (LH) promote OSE cell survival and rapid growth and facilitate entry into the carcinogenesis process. During these processes, molecular anti-apoptotic events are important for tumor establishment. Our previous study confirmed that FSH inhibits ovarian cancer apoptosis by upregulating survivin and downregulating the programmed cell death gene 6 (PDCD6) and death receptor 5 (DR5) (1). However, the actual role of FSH in OEC apoptosis is not yet fully understood.

OCT4, a member of the POU family of transcription factors, plays an important role in the maintenance of pluripotency and proliferation in embryonic stem cells. Indeed, it has been detected in a broad spectrum of cancers, including non-small lung cancer, hepatoma, breast cancer and bladder cancer (2–7). Moreover, as a stem marker, OCT4 is also expressed in cancer stem cells (CSC) or cancer stem cell-like cells (CSCLC), a minor population in tumor cells with specific features, such as self-renewal and reproducible tumor phenotype (8). A recent study identified that OCT4 and Nanog, another stem cell marker, are present in high-grade lung adenocarinoma (LAC) and can be used as markers of poor prognosis. The overexpression of OCT4 and Nanog in LAC was shown to increase the percentage of CD133-expressing subpopulation, sphere formation and induce CSC-like properties, implying that increasing OCT4 may, at least partly, enhance the CSCLC population by activating some stem cell pathways (9). However, unlike the majority of cells within the tumor, CSCs or CSCLCs are resistant to chemotherapy, which may be attributed to their anti-apoptotic properties. By small interfering RNA (siRNA) knockdown of OCT4, a previous study documented that the apoptosis of CSCLCs is mediated through the OCT4-TCL1-AKT pathway (10). In addition, hormones, such as estrogen and progesterone can augment mammary stem cells, which profoundly influence breast cancer risk (11,12). However, whether FSH-inhibited apoptosis well-defined by our previous study (1) is associated with increased OCT4 expression and activated stem cell signal pathway has not been clearly illustrated.

Ovarian CSCs or CSCLCs have been defined and isolated from ovarian cancer cell lines or peritoneal fluid from ovarian cancer patients using different markers, including CD133, CD44, CD117, Myd88 and ABCG2 (8,13–19). Among these markers, the CD44^+^CD117^+^ immunophenotype holds promise as a defining characteristic of ovarian CSCs or CSCLCs, since the isolated cells exhibit stem cell functionality. As these cells possessing stem cell properties also express OCT4, it is reasonable to hypothesize that this molecule may play a role in expansion of ovarian CSCLCs and activation of stem cell pathway.

Therefore, our objective in this study was to examine the relationship among FSH treatment, OCT4 expression and apoptosis. We detected OCT4 expression in a range of ovarian cancer tissue types and cell lines. The effects of FSH administration on OCT4 expression, apoptosis and expansion of the CD4^+^CD117^+^-expressing subpopulation in cancer cells were also investigated. Finally, the involvement of the FSH-OCT4-AKT-survivin pathway in apoptosis inhibition of ovarian cancer cells was explored.

## Materials and methods

### Clinical sample selection, tissue handling and pathologic analysis

All evaluated ovarian tissue samples were derived from the Department of Pathology at the First People’s Hospital (Shanghai Jiao Tong University, Shanghai, China). A total of 159 ovarian tissues specimens were studied. The formaldehyde-fixed and paraffin-embedded tissue specimens from 30 cases of benign ovarian cystadenomas, 30 cases of borderline tumors and 99 cases of ovarian carcinomas were collected between January 2003 and December 2010. Patients with a known history of hormone replacement, of prior radiation or chemotherapy were excluded. Pathological diagnoses of the above ovarian lesions were made by two gynecological pathologists based on the World Health Organization classification.

### OCT4 immunohistochemical staining and evaluation

Immunohistochemical analysis for OCT4 protein expression was performed as described previously (20). Briefly, OCT4 expression was detected using a rabbit polyclonal anti-human OCT4 IgG (ab19857; Abcam, Cambridge, UK). The sections were incubated with anti-OCT4 (1:200 dilution) in a moisture chamber for 2 h followed by a 45-min incubation with biotinylated secondary antibody. A section of spermatogonia was included in every experiment as a positive control. A rabbit IgG not against OCT4 was used as a negative control. The percentage of positively stained cells and the intensity of the staining in these slides were assessed in a blinded manner. OCT4 reactivity was graded on a score ranging from 0 to 12 based on the product of staining intensity (0 to 3) and percentage (≤5% scored 0, 6–25% scored 1, 26–50% scored 2, 51–75% scored 3, and >75% scored 4) of the cells stained. The staining intensity was graded for both on the following scale: 0, no staining; 1, weak staining; 2, moderate staining; and 3, intense staining. Individual IHC score for each case was used for statistical analysis. All IHC slides were reviewed independently by two investigators.

### Cell lines and culture

Ovarian cancer cell lines Hey, OVCAR-3, ES-2, HO8910, HO8910PM and A2780 were obtained from the American Type Culture Collection (Manassas, VA, USA) and cultured based on the guidelines of the repository. These cells were maintained in DMEM/F12 supplemented with 10% fetal bovine serum (FBS). The MCV152 and Moody cell lines kindly provided by Dr Wenxin Zheng (Arizona University, Tucson, AZ, USA) (21,22). SKOV3 and SKOV3-tax cells were purchased from the Beijing Union Medical College (Beijing, China) and were cultured in DMEM supplemented with 10% FBS, 100 U/ml penicillin, 100 *μ*g/ml streptomycin, sodium pyruvate and L-glutamine.

### Immunoblot analysis

Immunoblot analysis was performed as described previously (20). Briefly, ovarian cancer cells were treated with FSH at indicated concentrations and for indicated periods of time. The treated cells were lysed with RIPA buffer (50 mM Tris-HCl pH 7.4, 150 mM NaCl, 1% NP-40, 0.5% dexycholic acid, sodium, 0.1% SDS). Lysates were loaded on a 10% SDS-PAGE, transferred to polyvinylidene fluoride (PVDF) membranes and blocked in 5% non-fat milk in 10 mM Tris, pH 7.5, 100 mM NaCl and 0.1% (w/v) Tween-20 for 2 h. Proteins of interest were incubated with corresponding primary antibodies overnight at 4°C. Anti-β-actin or anti-GAPDH mouse monoclonal antibody (Lab Vision) was diluted at 1:1,000 for sample loading control. After washing three times with washing buffer (0.1% Tween-20 in TBS) and incubated with the appropriate secondary antibody at room temperature for 1 h, the membranes were washed and the bands visualized by enhanced chemiluminescence.

### Immunocytochemistry assay

Hey cells were plated in 6-well plates with coverslips for 24 h. The cells were then starved for 24 h in serum-free media and treated with 50 mIU/ml FSH for another 48 h. After washing with PBS three times, the cells were fixed for 10 min with formaldehyde and then permeabilized in 0.1% Triton X-100 in PBS for 5 min. Subsequently, the cells were incubated with an anti-human OCT4 primary antibody (diluted 1:100) at 37°C for 1 h, followed by rinsing three times in PBS for 5 min each time. After incubation with a FITC-labeled secondary antibody for another hour at room temperature, the cells were photographed.

### Total RNA extraction and RT-PCR analysis

Total RNA was isolated according to the protocol of the RNeasy Micro Kit (Qiagen, Frankfurt, Germany), and 2 *μ*g RNA was used for reverse transcription. The cDNA was then subjected to PCR amplification with primers list in Table I using the following conditions: initial denaturation at 95°C for 5 min, followed by 35 cycles of denaturation at 94°C for 30 sec, annealing at temperature listed in Table I for 30 sec, and extension at 72°C for 30 sec. The amplification products were detected on a 1% agarose gel with ethidium bromide staining. Relative mRNA levels of target genes were normalized to *GAPDH* which served as a loading control.

### Apoptosis assay

Cells undergoing various treatments were harvested, fixed in 70% ethanol overnight at 4°C and stained with 50 mg/ml propidium iodide. The Annexin V-FITC Apoptosis Detection kit was used to identify apoptotic and viable cells following the manufacturer’s instructions. The populations of early apoptotic cells were detected by flow cytometry.

### Isolation of ovarian CSCLCs

To evaluate the effect of FSH on ovarian CSCLC expansion, flow cytometry was used to determine the changes in populations of CSCLCs. Seven ovarian cancer cell lines were treated with 50 mIU/ml FSH for 48 h, then the treated and untreated cells were collected and double labeled with antibodies against CD44 (conjugated with FITC) and CD117 (conjugated with phycoerythrin) (BD Systems, Minneapolis, MN, USA). Labeled cells were detected with a FACSCalibur (Becton-Dickinson Immunocytometry Systems, San Jose, CA, USA).

### Transient transfection of OCT4 siRNA and hormone treatment

Hey and OVCAR-3 cells were plated in 6-cm dishes at a density of 2×10^4^ cells/ml. After 24 h of culture, the medium was replaced by Opti-MEM (Invitrogen, Carlsbad, CA, USA) in the absence of antibiotics and cultured for another 24 h. siRNA corresponding to the OCT4 gene was designed and synthesized by Dharmacon (Thermo Scientific), and the sequences of SMARTpool siRNA against OCT4 included: 5′-GCGAUCAAGCAGCGACUAU-3′, 5′-UCCCAUGCAUUCAAACUGA-3′, 5′-GCACUGUACU CCUCGGUCC-3′, 5′-CGAGAAGGAUGUGGUCCGA-3′. These siRNA were transiently transfected into the cells with DharmaFECT transfection reagents (Thermo Scientific) according to the manufacturer’s instructions. After incubation for another 24 h, a portion of the cells was collected for RNA extraction and semi-quantitative RT-PCR analysis to determine the degree of gene silencing and detect the effect of knocking down OCT4 on *Sox2*, *Notch* and *Nanog* gene expressions in the absence or presence of FSH. The other portion of the treated cells was used to investigate the effect of OCT4 depletion on downstream proteins by western blot analysis in the absence or presence of FSH.

### Plasmid construction and transfection

The OCT4 ORF was inserted into the *Eco*RI-*Bam*HI site of pIRES2-EGFP to generate the pIRES2-EGFP-OCT4 plasmid. The integrity of the cDNA was confirmed by sequencing (data not shown). To investigate the effect of OCT4 overexpression on *Sox2*, *Notch* and *Nanog* genes, 4 *μ*g pIRES2-EGFP-OCT4 and 4 *μ*g empty vector were transfected into Hey and OVCAR-3 cells using Lipofectamine 2000 (Invitrogen) according to the instructions provided by the manufacturer. The changes in *Sox2*, *Notch* and *Nanog* gene transcripts were determined by RT-PCR. *GAPDH* served as a loading control.

### Production of lentiviral particles

In order to prepare lentiviral particles expressing the *OCT4* gene, HEK-293T cells were transfected with pLenti-OCT4 (OCT4 ORF was constructed into modified pLOV.CMV.eGFP.EF1a.PuroR which was removed the eGFP gene) plus lentiviral packaging vectors. Briefly, the cells were seeded in a 6-well plate at a concentration of 1.0×10^6^ cells per well. After 24 h, the culture medium was aspirated and replaced with Opti-MEM (Invitrogen). Subsequently, 2 *μ*g pLenti-OCT4 and the Mission Lentiviral Packaging mix (Sigma-Aldrich, St. Louis, MO, USA) were transfected into cells by Lipofectamine 2000 (Invitrogen) according to the manufacturer’s instructions. The following day, the culture was replaced with complete medium (DMEM with 10% FBS). After another 48 h, the culture supernatants containing the lentiviral particles (Lenti-OCT4) were harvested for use.

### Establishment of OCT4-overexpressing ovarian cancer stable cell lines

To establish the OCT4-overexpressing ovarian cancer cells, Hey cells were plated in 6-well plates with 2.0×10^5^ cells per well and transduced with the Lenti-OCT4 lentiviral particles. On the third day after transduction, puromycin (Sigma-Aldrich) was added into the culture medium to a final concentration of 2 *μ*g/ml. During the selection period, the drug was kept at the same concentration at each replacement of culture medium. Approximately 2 weeks was required for the live cells to be eliminated in the mock transduction group. After that, the selected cultures were expanded and cryopreserved. The OCT4 mRNA and protein expressions were detected by PCR and western blot analysis, respectively.

### Statistical analyses

The statistical significance of the differences in the immunohistochemical staining in ovarian tissues was calculated using the χ^2^ test. The differences of OCT4 expression in ovarian cancer tissues grouped by age, grade, stage, lymph node metastasis and menopause status was investigated using the χ^2^ test. A two-sided test with P<0.05 was considered statistically significant. All statistical analysis was performed using SPSS 11.0 (SPSS Inc., Chicago, IL, USA) or Prism 5.0 (GraphPad Software).

## Results

### Ovarian carcinoma overexpress OCT4 protein

**Figure 1.**
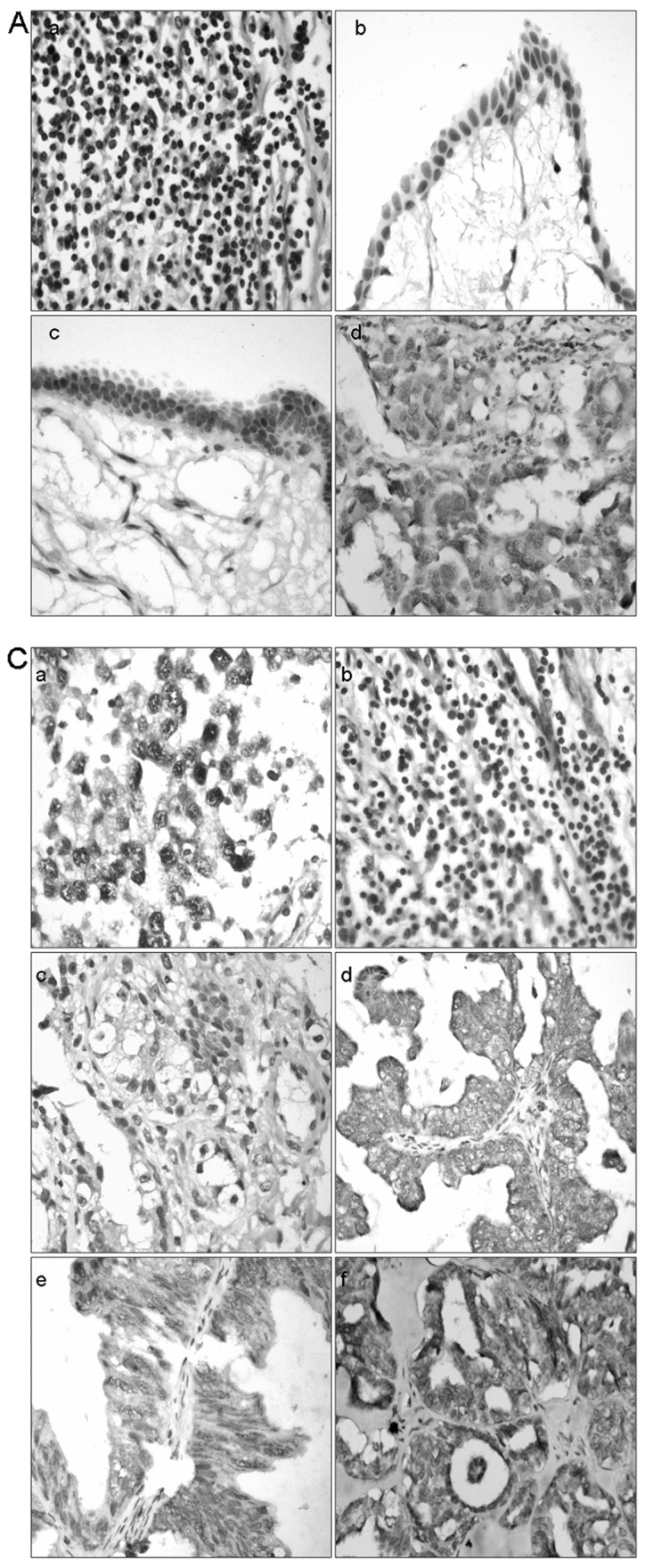

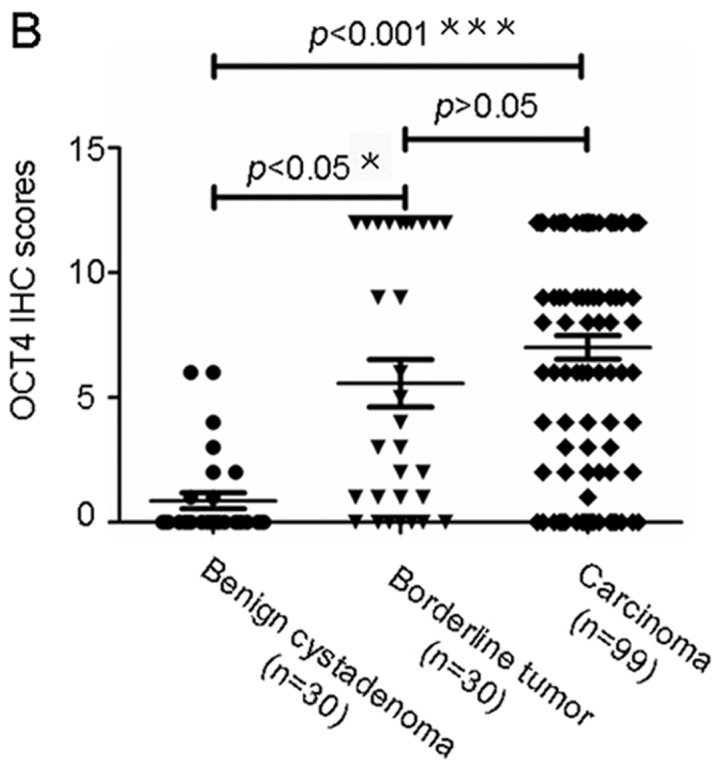

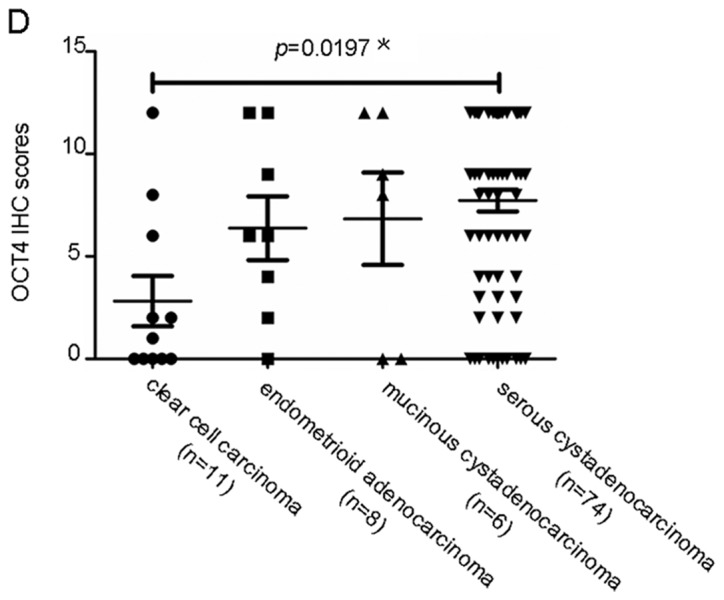
OCT4 protein expression in ovarian tissues. (A) IHC staining of ovarian tissues for OCT4 protein expression. Tissues were stained with a polyclonal OCT4 antibody. (a) Few cells expressing OCT4 were detected in (b) benign cystadenoma whereas positive staining of OCT4 was observed in (c) borderline tumors and (d) ovarian carcinoma. (B) Comparison of OCT4 protein among benign cystadenoma, borderline tumors and ovarian carcinoma tissues. ^***^P<0.001, statistically significant compared with benign cystadenoma. ^*^P<0.05, compared with benign cystadenoma. (C) Different expression patterns of OCT4 in OEC tissues with different pathological types. Spermatogonia stained with (a) a polyclonal OCT4 antibody or (b) a rabbit IgG not against OCT4 serve as positive or negative control, respectively. Variable OCT4 expression was examined in (c) clear cell carcinoma, (d) endometrioid adenocarcinoma, (e) mucinous cystadenocarcinoma and (f) serous cystadenocarcinoma. (D) Comparison of OCT4 protein expression among different pathological types OEC tissues. ^*^P<0.05, compared with clear cell carcinoma.

Ovarian tissue samples from 159 patients were used in this study, and the expression of OCT4 was analyzed using immunohistochemical (IHC) staining. As shown in Table II, 2 of 30 cases (6.7%) of benign cystadenomas had high levels of OCT4 protein, whereas 14 of 30 cases (46.7%) of borderline tumors and 65 of 99 cases of carcinoma (65.7%) showed high expression of OCT4 protein. Significantly increased OCT4 was observed in borderline tumors when compared with benign cystadenomas. Representative images are shown in Fig. 1A. Low expression of OCT4 in nuclei could be seen in benign cystadenomas (Fig. 1Ab), whereas positive staining of OCT4 localized in nuclei in borderline tumors and in cytoplasm in carcinomas (Fig. 1Ac and Ad). Spermatogonia and OEC samples cultured with IgG not against OCT4 served as a negative control (Fig. 1Aa). Further study revealed different expression profiles of OCT4 in different OEC pathological types (Fig. 1C). Low levels of OCT4 protein were detected in 8 of the 11 clear cell carcinomas (72.7%); however, high levels of OCT4 protein were examined in 5 of the 8 endometrioid adenocarcinoma (62.5%), 4 of the 6 mucinous cystadenocarcinoma (66.7%) and 53 of the 74 (71.6%) serous cystadenocarcinoma cases (Table III and Fig. 1Cc–f). Oct4 expression was scored as high or low for each case according to the criteria described in Materials and methods, and the data are summarized in Fig. 1B and D. The correlations between OCT4 expression and clinical pathological factors in serous cystadenocarcinoma were further investigated. We found that OCT4 expression was significantly associated with histological grade (P=0.008) but not with age (P=0.611), clinical stage (P=0.954), lymph node metastasis (P=0.402) or menopause (P=1.0) (Table IV). These results suggested OCT4 may play a role in epithelial ovarian cancer.

### FSH upregulates OCT4 expression

**Figure 2.**
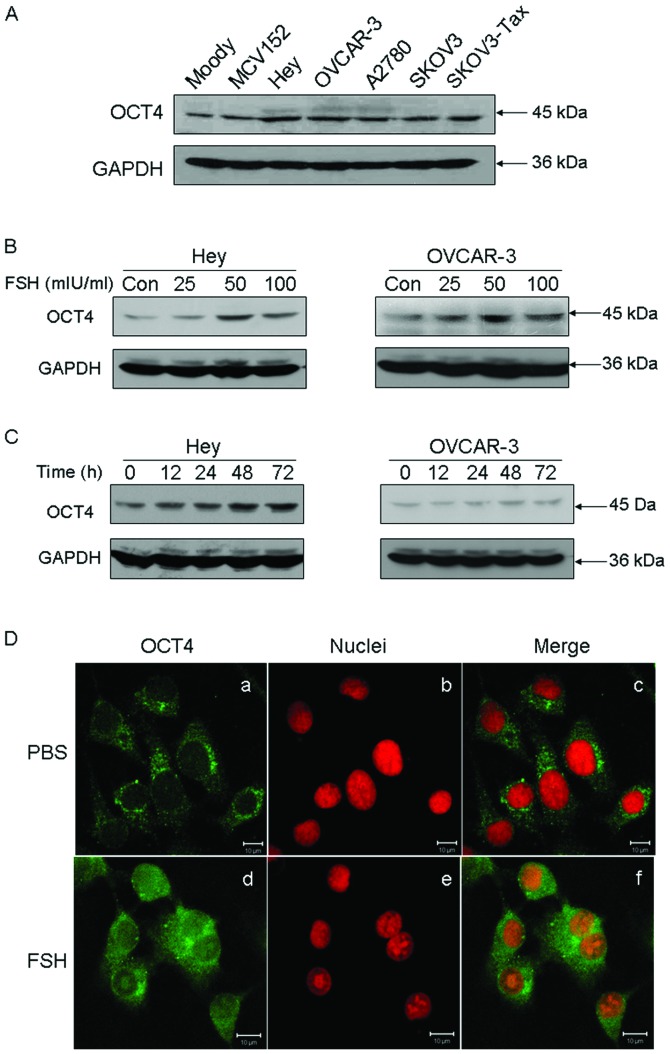
FSH upregulates OCT4 expression in ovarian cancer cell lines. (A) OCT4 protein expression in immortalized OSE cells (Moody cells) and ovarian cancer cell lines. GAPDH served as a loading control. (B) FSH elevated OCT4 expression in a dose-dependent manner in Hey and OVCAR-3 cells after 48 h treament. (C) FSH (50 mIU/ml) increased OCT4 expression in a time-dependent fashion in Hey and OVCAR-3 cells. (D) Immunofluorescence assay of OCT4 protein expression and localization in Hey cells after 50 mIU/ml FSH treatment for 48 h.

Extensive studies during the last decade have provided strong evidence that OCT4 is expressed in various cancer types, including lung cancer, prostate cancer, liver cancer and cervical cancer (3,4,9,23–25). In the current study, the expression of OCT4 was determined by western blot analysis in OET cells. As shown in Fig. 2A, OCT4 was found to be overexpressed in ovarian cancer cells, compared with the normal Moody ovarian cell line and the SV40-transformed benign ovarian MCV152 cell line.

FSH is a confirmed risk factor for OEC. In order to evaluate the relationship between FSH and OCT4 overexpression in ovarian cancer tissues, the effect of FSH on OCT4 expression was studied. As shown in Fig. 2B, FSH stimulation resulted in a significant increase in OCT4 protein in a dose-dependent manner in both Hey and OVCAR-3 cell lines. The most significant upregulation of OCT4 was observed when cells were exposed to 50 mIU/ml FSH for 48 h. Moreover, FSH was found to enhance OCT4 expression with a time-dependent manner (Fig. 2C). Subcellular analysis by immunofluorescence staining confirmed that FSH stimulation increased OCT4 protein expression in both the cytoplasm and nucleus in Hey cells (Fig. 2D).

### FSH inhibits apoptosis in OEC cells

**Figure 3.**
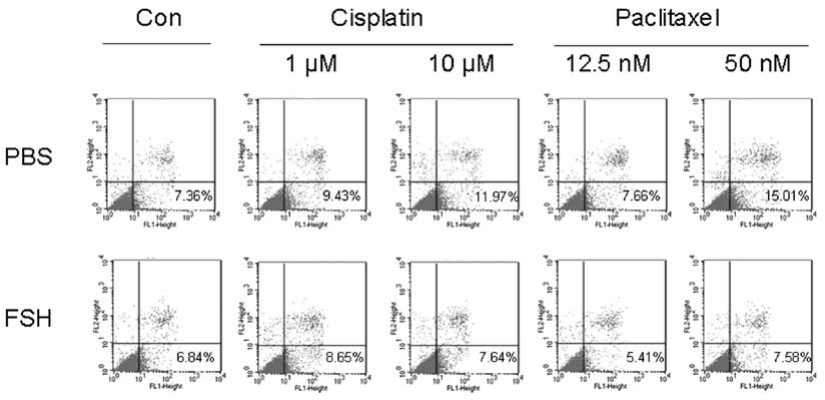
FSH inhibits ovarian cancer cell apoptosis. FSH attenuated the dose-dependent induction of apoptosis by chemotherapy drugs in Hey cells.

The overexpression of OCT4 protein in ovarian cancer tissue implies that it is involved in OET development. Indeed, it was previously confirmed that depletion of OCT4 in lung cancer cells would result in CSCLC apoptosis (10). Although we showed positive regulation of OCT4 expression by FSH treatment, whether FSH has a direct effect on ovarian cancer cell apoptosis requires further exploration. We found that treatment with the chemotherapeutic drugs cisplatin or paclitacel at various concentrations could induce early apoptosis in a dose-dependent manner; however, in the presence of FSH, the induced apoptosis rates were partly abrogated in Hey (Fig. 3) cells. We thus concluded that FSH is involved in apoptosis inhibition in ovarian cancer cells.

### Apoptosis induced by knockdown of OCT4 is attenuated by FSH

**Figure 4.**
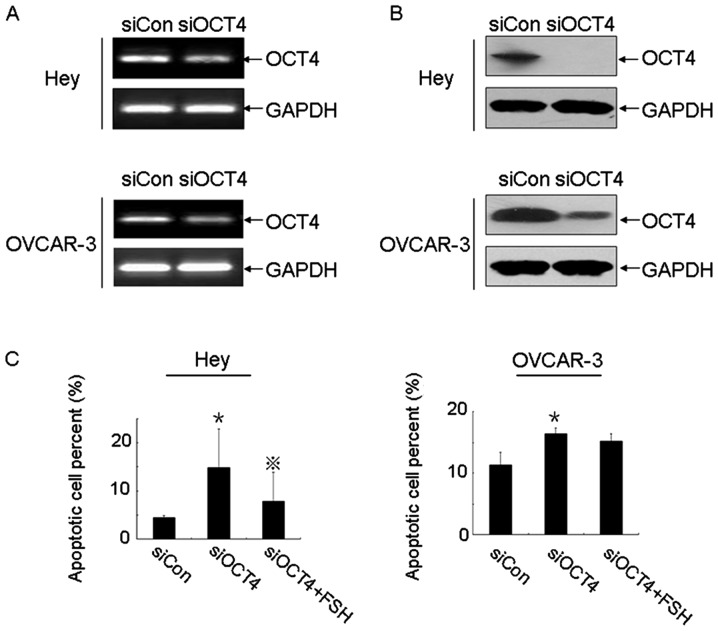
Knockdown of OCT4 induces ovarian cancer cell apoptosis and FSH attenuates this effect. (A) RT-PCR examination of *OCT4* mRNA expression after siRNA mediated knockdown in Hey and OVCAR-3 cells. (B) Western blot analysis of OCT4 protein expression after knockdown in Hey and OVCAR-3 cells. (C) Depletion of OCT4 induced apoptosis, and FSH administration attenuated this effect. Each experiment repeated three times. ^*^P<0.05, compared with siCon; ^※^ P<0.05, compared with siOCT4.

In order to determine whether OCT4 plays a role in FSH-induced inhibition of apoptosis, OCT4 was knocked down by siRNA. As shown in Fig. 4A and B, OCT4 mRNA and protein levels were potently reduced after depletion of OCT4. Moreover, knocking down OCT4 resulted in a significantly increased early apoptosis rate in Hey and OVCAR-3 cells. However, in the presence of FSH (50 mIU/ml), this early apoptosis rate induced by OCT4 knockdown was decreased in both cell lines, although marked changes were not observed in OVCAR-3 cells (Fig. 4C).

### FSH induces ovarian CSCLC expansion

**Figure 5.**
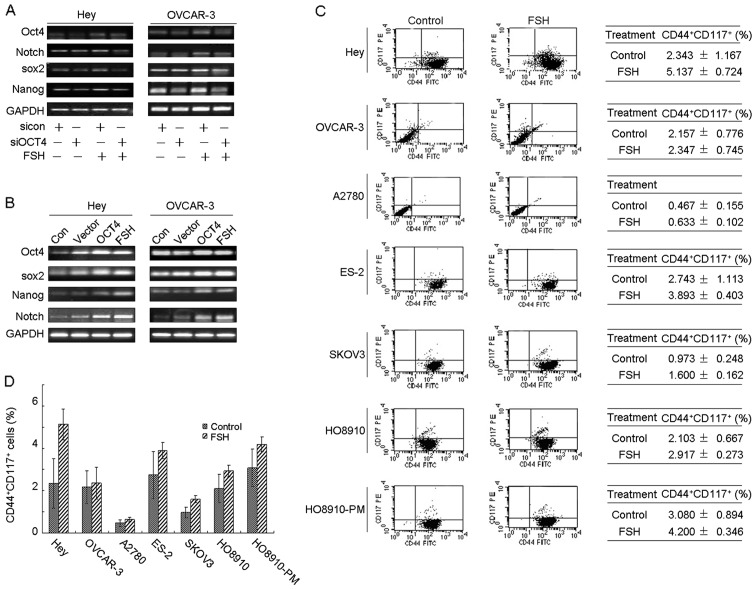
FSH enhances expression of stem cell markers and induces ovarian CSCLC expansion. (A) Knockdown of OCT4 reduced *Notch*, *Sox2* and *Nanog* mRNA expression and blocks FSH induction of these genes. (B) Overexpression of OCT4 increased *Notch*, *Sox2* and *Nanog* gene expression. (C) FSH induced ovarian CSCLC expansion. (D) Comparison of FSH induced ovarian CSCLC populations among ovarian cancer cell lines.

Although *OCT4*, *Sox2*, *Nanog* and *Notch* are all important stem cell markers, the relationships between them are not clear. Here we studied changes in other CSC markers while modulating OCT4 expression. As indicated in Fig. 5A, depletion of OCT4 using siRNA resulted in reduction of *Notch*, *Sox2* and *Nanog* mRNA. FSH induced *Notch*, *Sox2* and *Nanog* expression were abolished by knocking down OCT4. Similar patterns were obtained in both Hey and OVCAR-3 cells. By contrast, upregulated *Sox2*, *Nanog* and *Notch* mRNA were detected after transient transfection with the pIRES2-EGFP-OCT4 plasmid, whereas transfection with empty vector had no effect on the expression of these genes (Fig. 5B). Similar to the effect of pIRES2-EGFP-OCT4 transfection in OEC cells, FSH stimulation also potently enhanced the expression of these CSC markers (Fig. 5B). These data suggest that FSH may play a role in regulating CSC marker expression via OCT4 mediated stem signal pathway. Although these markers positively responded to FSH treatment, whether FSH increases the population of CSCLCs remained to be clarified. To investigate the changes in a broad range of ovarian CSCLCs, we used Hey, OVCAR-3, A2780, ES-2, SKOV3, HO8910 and HO8910PM cell line to detect the population of CD44^+^CD117^+^ cells after 50 mIU/ml FSH stimulation for 48 h. The results indicated that FSH induced expansion of the CD44^+^CD117^+^ cell population, especially in the Hey and ES-2 cell lines (Fig. 5C and D), which implies that OCT4 mediated stem signal pathway may involve inhibition of FSH-induced apoptosis.

### Stable transfection of OCT4 increases CSCLCs and inhibits apoptosis

**Figure 6.**
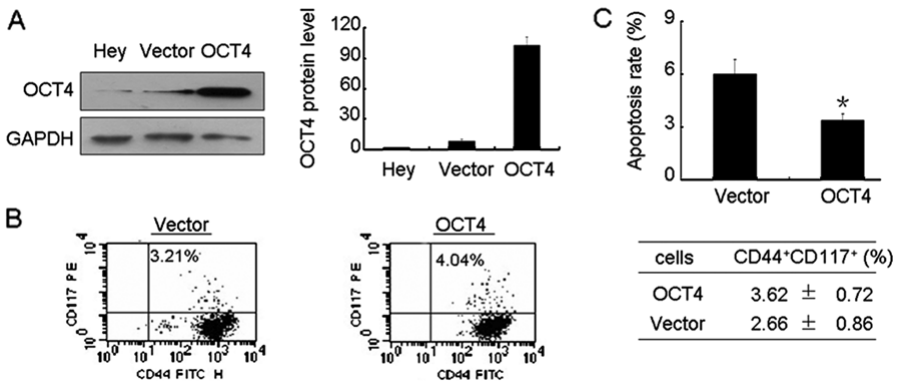
Overexpression of OCT4 increases the proportion of ovarian CSCLCs and inhibits ovarian cancer cell apoptosis. (A) Western blot analysis of OCT4 expression in OCT4 stable transfected cells, vector transfected cells and parental Hey cells. (B) Overexpression of OCT4 induced ovarian CSCLC expansion in Hey cells. (C) Overexpression of OCT4 inhibited ovarian cancer cell apoptosis. ^*^P<0.05, compared with the vector group.

To further investigate the role of FSH-elevated OCT4 in ovarian cancer apoptosis, we stably overexpressed OCT4 in Hey cells as described in Materials and methods. As shown in Fig. 6A, a high level of OCT4 protein was observed in OCT4 transfected Hey cells. Moreover, elevated OCT4 resulted in an increased level of CD44^+^CD117^+^ cells (Fig. 6B). Further study found that elevated OCT4 significantly reduced the apoptosis rate compared with Hey cells transfected with empty plasmid (Fig. 6C).

### OCT4-AKT-survivin pathway is involved in FSH mediated inhibition of apoptosis

**Figure 7.**
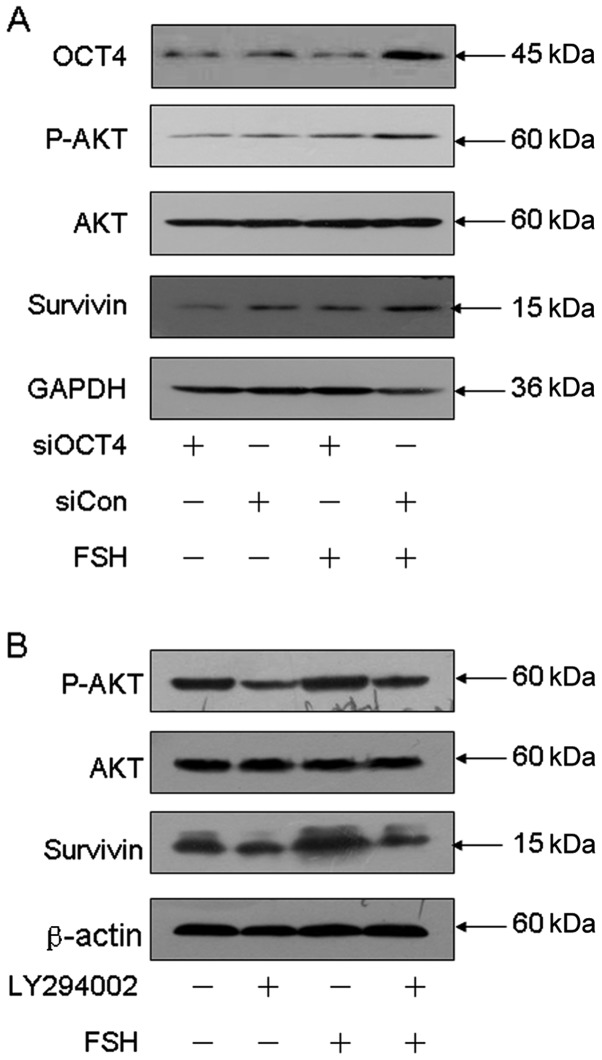
Potential involvement of the OCT4-AKT-survivin pathway in inhibition FSH-induced apoptosis. (A) Depletion of OCT4 abolished FSH-upregulated survivin and activated AKT. (B) Inactivation of AKT blocked FSH-induced expression of survivin.

Our previous work confirmed that survivin regulates apoptosis in ovarian cancer and endometrial cancer cells. Moreover, the OCT4-AKT-ABCG2 and OCT4-TCL1-AKT signaling pathways were demonstrated to participate in chemo-resistance and apoptosis inhibition. Therefore, we investigated the relationships between OCT4, AKT and survivin. As shown in Fig. 7A, depletion of OCT4 resulted in reduction of p-AKT and survivin, and also attenuated FSH-induced p-AKT and survivin protein levels. Furthermore, chemical inhibition of AKT using LY294002 abolished the FSH mediated elevation of survivin. These data indicated that FSH induces ovarian cancer apoptosis through the OCT4-AKT-survivin signaling pathway.

## Discussion

Epithelial ovarian cancer is a common gynecological cancer worldwide, especially in postmenopausal women or individuals who have received treatment to induce ovulation (26–28). Prior studies have confirmed that the enriched hormonal environment of the ovary influences the development of OEC (27,29). Elevated gonadotropins in postmenopausal women, especially FSH, have been hypothesized to contribute to the incidence of OEC. Indeed, there are considerable lines of evidence showing that FSH promotes ovarian cancer cell proliferation and invasion (30–32). Our recent study implicated an anti-apoptotic effect of FSH in OEC development (1). It has also been demonstrated that chemoresistant hepatocellular carcinoma cells are enriched for CSCs and that the OCT4-AKT-ABCG2 pathway acts on CSCs to promote cell proliferation through inhibition of apoptosis (25). Therefore, whether the apoptosis inhibition in ovarian cancer cells result from FSH-induced stem cell related signal pathway remains obscure.

In the current study, we investigated OCT4 as a potential stem cell and CSC marker for OEC. OCT4 protein expression was measured in one hundred fifty-nine cases of different types of ovarian lesions. The results of IHC staining indicated that OCT4 expression was higher in ovarian carcinomas and borderline tumors, while it was marginally expressed in benign cystadenomas (Fig. 1A). Similar expression patterns were also observed in a normal ovarian epithelial cell line and ovarian cancer cells (Fig. 2A). Moreover, we found a statistically significant association of OCT4 expression with ovarian carcinomas of different pathological types. A relatively low level of OCT4 expression was found in clear cell carcinoma, while a large percent of serous carcinoma had a higher level of OCT4 when compared with endometrioid adenocarcinoma and mucinous cystadenocarcinoma, all of which showed positive staining for OCT4 (Fig. 1C). This finding is consistent with previous studies showing that a high level of OCT4 is usually present in poor prognosis patients (24,33). To our knowledge, this is the first large-scale study of OCT4 expression in ovarian lesions.

To further investigate the relationship between elevated OCT4 expression and the development of OEC, determination of the effect of FSH, as a high risk factor for OEC incidence, on OCT4 expression was required. Our results clearly indicated that FSH markedly enhanced OCT4 expression in both the cytoplasm and nucleus (Fig. 2B–D). This result prompted us to further investigate the role of FSH-induced OCT4 in OEC development. In previous reports, depletion of OCT4 was shown to result in apoptosis and cell growth arrest (10,34), whereas OCT4 overexpression enhances chemotherapy resistance in liver cancer (25). In this study, we found that FSH attenuated ovarian cancer cell apoptosis induced by chemo-therapeutic drugs (Fig. 3). Further investigation revealed that FSH abolished depletion of OCT4 induced-apoptosis (Fig. 4). These data suggest that OCT4 participates in FSH-induced apoptosis inhibition. However, the effects of changes of OCT4 on the expression and proportionality of other CSCs or CSCLCs are not clear. Chiou *et al* reported that co-expression of OCT4 and Nanog, another stem cell marker, in lung adenocarcinomas can increase the proportion of CD133-expressing subpopulation, sphere formation and enhance drug resistance (9). In ovarian cancer, Zhang *et al* identified CD44^+^CD117^+^ cells as ovarian CSC-like cells (8). In the current findings, it was confirmed that the cells stably transfected with OCT4 had a slightly increased level of the CD44^+^CD117^+^ subpopulation (Fig. 6B) and markedly induced inhibition of cellular apoptosis (Fig. 6C), implying that stem signal pathway may involve apoptosis inhibition. Since FSH upregulated OCT4 expression, the elevated OCT4 may mediate FSH-induced apoptosis inhibition. As expected, most ovarian cancer cell lines showed an increase in the CD44^+^CD117^+^ subpopulation (Fig. 5C and D) after FSH treatment. It was observed that FSH upregulated *Notch*, *Sox2* and *Nanog* mRNA, and this effect was blocked by knocking down OCT4. Thus, FSH-induced stem signal related signal pathway may be another mechanism of FSH related apoptosis inhibition in ovarian cancer cells.

Previous studies showed that the OCT4-TCL1-AKT and OCT4-AKT-ABCG2 signaling pathways are involved in CSC proliferation, chemoresistance and apoptosis (10,25). As we have found in prior studies that survivin participates in inhibition of apoptosis in various cancer cells (1,20,35,36), we investigated the relationship between OCT4 and survivin. Our data clearly showed that knockdown of OCT4 decreased AKT activation and reduced survivin expression. FSH-induced activation of AKT and elevated survivin were abolished by depletion of OCT4 (Fig. 7A), while blockage of AKT signaling inhibited FSH-induced survivin (Fig. 7B). This finding indicates that FSH inhibits apoptosis through the OCT4-AKT-survivin signal pathway in OEC cells.

In conclusion, we showed that OCT4 may play a role in OEC development as it was overexpressed in human ovarian carcinomas compared with benign cystadenomas. OCT4 expression was also associated with the tumor grading, with higher levels indicating poor prognosis. We further found that elevated OCT4 contributes to FSH-induced apoptosis inhibition through OCT4-AKT-survivin signal pathway. Our findings provide novel insights into FSH inhibition of ovarian cancer apoptosis.

## Figures and Tables

**Table I.** Sequences of primers used for amplification of target genes.

Gene	Primer sequence (5′→3′)	Annealing temp. (°C)
*OCT4*	F: GTACTCCTCGGTCCCTTTCC	58
	R: CAAAAACCCTGGCACAAACT	
*Sox2*	F: GGAGCTTTGCAGGAAGTTTG	60
	R: GGAAAGTTGGGATCGAACAA	
*Nanog*	F: TTGGAGCCTAATCAGCGAGGT	58
	R: GCCTCCCAATCCCAAACAATA	
*Notch*	F: CAACATCCAGGACAACATGG	60
	R: GGACTTGCCCAGGTCATCTA	
*GAPDH*	F: AACGGATTTGGTCGTATTG	56
	R: GGAAGATGGTGATGGGATT	

F, forward; R, reverse.

**Table II.** Expression of OCT4 in distinct tumor tissue types.

Tissue type	Total	High level	Low level	%	P-value
Cystadenomas	30	2	28	6.7	<0.0001
Borderline tumor	30	14	16	46.7	
Carcinomas	99	65	34	65.7	

IHC score less than 4 was considered as low expression; 5–12 as high expression. %, percentage of high level OCT4 expression. χ^2^ test, P<0.0001.

**Table III.** Expression of OCT4 in different OEC pathological types.

Pathological type	Total	Expression		%	P-value
		High	Low		
Clear cell carcinoma	11	3	8	27.3	0.039
Endometrioid adenocarcinoma	8	5	3	62.5	
Mucinous cystadenocarcinoma	6	4	2	66.7	
Serous cystadenocarcinoma	74	53	21	71.6	

%, percentage of high level OCT4 expression. χ^2^ test, P=0.039.

**Table IV.** Correlations of OCT4 expression and major clinical pathologic factors in serous cystadenocarcinoma (n=74).

Factors	No. of patients	OCT4 expression		P-value
		High	Low	
Age (year)				0.611
≥55	38	26	12	
<55	36	27	9	
Histological grade				0.008
Low (I)	26	13	13	
Intermediate (II)	24	19	5	
High (III)	24	21	3	
Clinical stage				0.954
I	8	6	2	
II	15	11	4	
III	51	36	15	
Lymph node metastasis				0.402
No	31	24	7	
Yes	31	20	11	
Menopause				1.000
Yes	42	30	12	
No	15	11	4	

## Abbreviations:

CSCLC: cancer stem cell-like cell;
CSC: cancer stem cells;
FSH: follicle stimulating hormone;
OET: ovarian epithelial tumor;
OEC: ovarian epithelial cancer
